# Platinum-coated silicotungstic acid-sulfonated polyvinyl alcohol-polyaniline based hybrid ionic polymer metal composite membrane for bending actuation applications

**DOI:** 10.1038/s41598-022-08402-x

**Published:** 2022-03-16

**Authors:** Mohammad Luqman, Hamid Shaikh, Arfat Anis, Saeed M. Al-Zahrani, Abdullah Hamidi, Inamuddin

**Affiliations:** 1 Department of Chemical Engineering, College of Engineering, Taibah University, Yanbu, Saudi Arabia; 2 SABIC Polymer Research Centre, Department of Chemical Engineering, King Saud University, P.O. Box 800, Riyadh, 11421 Saudi Arabia; 3 Department of Applied Chemistry, Zakir Husain College of Engineering and Technology, Faculty of Engineering and Technology, Aligarh Muslim University, Aligarh, 202002 India

**Keywords:** Chemistry, Energy science and technology, Engineering, Materials science, Nanoscience and technology

## Abstract

An electro-stimulus-responsive bending actuator was developed by synthesizing a non-perfluorinated membrane based on silicotungstic acid (SA), sulfonated polyvinyl alcohol (SPVA), and polyaniline (PANI). The membrane was developed via solution casting method. The dry membrane SA/SPVA showed a sufficient ion-exchange potential of 1.6 meq g^−1^ dry film. The absorption capacity of the membrane after almost 6 h of immersion was found to be ca. 245% at 45 °C. The electroless plating with Pt metal was carried out on both sides of the membrane that delivered an excellent proton conductivity of 1.9 × 10^−3^ S cm^−1^. Moreover, the scanning electron microscopy (SEM) was conducted to reflect the smooth and consistent surface that can prevent water loss. The water loss capacity of the membrane was found to be ca. 33% at 6 V for 16 min. These results suggest a good actuation output of the ionic polymer metal composite **(**IPMC) membrane once the electrical potential is applied. The electromechanical characterization displayed a maximum tip displacement of 32 mm at 3 V. A microgripping device based on multifigure IPMC membrane may be developed showing a good potential in micro-robotics.

## Introduction

Recently, ionic polymer-metal composites (IPMCs) are evolving as one of the most promising electroactive active polymers (materials) (EAPs) that have received immense attention in various fields including robotic actuators, dynamic sensors, and artificial muscles^1–4^. These are serving dynamic applications owing to their noteworthy characteristics such as easy processing, light in weight, flexibility, high sensitivity, and resilience^5^. Interestingly, IPMC based actuators can function at very low external electrical stimuli ranging from 1–6 V and can work underwater and in the air^6^. It has been noticed that compared to the traditional robotic actuation materials, IPMCs show more significant bending deformation under biological (human) environment^7^. Therefore, IPMCs are being tried for their commercial utilization in many biomedical and/or biocompatible devices including implantable heart-assist and compression devices, virtual reality tactile displays, and biologically inspired robotic systems^7^.

Usually, the IPMCs consist of a newly trailed semi-permeable ion-exchange polymer (ionomer) membrane laminated on both sides with metal such as platinum (Pt) or gold (Au) as an electrode and water as an internal metal cation dissociation and transportation medium^8–12^. As a result, on application of the electric voltage, the polymer membrane expands near the cathode, causing a strain in the cation-rich region of the IPMC membrane that leads to bending in the direction of the anode. However, cross-linked cations are immobile under dry environments^13^. Conversely, cations are surrounded by water molecules in moist environments (due to hydration), making the entire film mobile^14^. Consequently, the mobility of ions in the IPMC film is due to the cations enclosed with water molecules. Various perfluorinated polymer ionomers are commercially available and used in IPMCs actuator applications due to their strong chemical and physical properties^15–20^. Generally, perfluorinated IPMC membranes with the Nafion trade name are commonly utilized as actuators and dynamic sensors owing to their inherent advantages, including fast proton exchange capacity and in terms of chemical, thermal, and mechanical stabilities^21^. Excessive water evaporation and the production cost of the Nafion membrane prompts researchers to find alternatives to these traditional membranes.

Replacing the conventional Nafion membranes with non-perfluorinated membranes may resolve the above mentioned issues^9,10,22–24^. Therefore, researchers are emphasizing to develop low-cost non-perfluorinated membranes with increased water retention even at high temperatures in addition to easy processing. Conventional ionic polymeric materials including styrene and vinyl alcohol have been modified (including sulfonation and/or carboxylation) during the preceding years as substitute for perfluorinated ionic polymers as bending actuators by scientists and engineers^9,10,24–28^.

Enhanced tip displacement, bending deformation, and thermal stability are all characteristics of these IPMCs. On the other hand, back relaxation is the most significant disadvantage of IPMC actuators that limits bending movement and acceptable frequency range. Also, excessive water loss from IPMC membranes in response to an applied electric potential generates quick performance degradation, back relaxation behavior, and extended processing time, which are vital to IPMC actuator issues.

Keeping issues in mind, in this study, a ternary composite that is composed of a non-perfluorinated sulfonated polyvinyl alcohol (SPVA), silicotungstic acid (SA), and polyaniline (PANI) based IPMC membrane was synthesized. After that, Pt coating was carried out using an electroless plating procedure over the synthesized membrane (SA/SPVA-PANI-Pt).

The aim of conducting polymer coating over a traditional IPMC actuator is to enhance the actuator's performance by reducing the creation of membrane surface fissures. The sulfonated nonperfluorinated ionic polymers have been used to prepare IPMC actuators that show prolong response times and back relaxation. To enhance the actuators’ properties, it was considered worthwhile to develop an SPVA–polyaniline (PANI) polymer membrane-based IPMC. As a consequence, the fabricated membrane (SA/SPVA-PANI-Pt) may serve as a convenient and dependable solution for the development of innovative actuators along with promising industrial applications.

## Experimental

### Materials

Polyvinyl alcohol (average molecular weight 85,000 to 1,24,000, Sigma-Aldrich Chemie Pvt. Ltd., USA), silicotungstic acid (Loba Chemie, India), and 4-sulfophthalic acid, 50 wt.% solution in water (Sigma-Aldrich Chemie Pvt. Ltd., USA). Aniline monomer (C_6_H_5_NH_2_) was procured by Thermo Fisher Scientific Pvt. Ltd., India, Potassium peroxydisulfate (K_2_S_2_O_8_; extrapure), and ammonium hydroxide (NH_4_OH; 25%) were obtained from Merck Specialties Pvt., Ltd., Germany. Tetraamineplatinum(II) chloride monohydrate Pt(NH_3_)_4_Cl_2_.H_2_O (Crystalline) (Alfa Aesar, USA), sodium borohydride (Thomas Baker Pvt. Ltd., India) and ammonium hydroxide (25%) (Merk Specialties Pvt. Ltd., India) and hydrochloric acid (HCl) obtained by Thermo Fisher Scientific Pvt. Ltd., India were used as received without further purification.

### Instruments

The Fourier transform infrared spectroscopy (FTIR; Nicolet iS50 FT-IR) was carried out in the range 4000–400 cm^−1^ to ascertain the functional group of the material. X-ray diffraction analysis (XRD; Rigaku Smart Lab X-ray diffractometer) was carried out to determine the crystallinity of the given membrane. Additionally, scanning electron microscopy (SEM; JSM, 6510 LV, JEOL, Japan) was operated at 200 kV on a copper grid coated by carbon paper. It was carried out to understand the surface morphology of the prepared membrane. Moreover, the elemental analysis was carried out using Energy dispersive x-ray (EDX) spectroscopy.

### Preparation of the reagent solutions

10 percent v/v C_6_H_5_NH_2_ and 0.1 M K_2_S_2_O_8_ solutions in 1 M HCl were prepared. Aqueous solutions of Pt(NH_3_)_4_Cl_2_.H_2_O (0.04 M), NH_4_OH (5.0 percent), and NaBH_4_ (5.0 percent) were made in demineralized water.

### Synthesis of cation exchanger material

The ionomer was prepared by dissolving 4 g of polyvinyl alcohol in 100 ml demineralized water followed by stirring up to 6 h at 60 ºC. After complete dissolution, a clear solution was obtained. Sulfonation was carried out by adding 4 ml of 4-sulfophthalic acid with constant stirring for 2 h at 60 ºC. A 1 g of silicotungstic acid was added into the sulfonated polyvinyl alcohol solution. Now the mixture was again stirred for 4 h at 50 ºC. The solution was homogenized for 12 h at room temperature and washed to remove the impurities and unreacted acid. The washing was carried out using Whatman filter paper and placed the membrane over it, kept over the funnel. After that, distilled water was passed gradually through it till the pH of the filtrate became neutral.

### Preparation of ionomeric membrane

**Figure 1:**
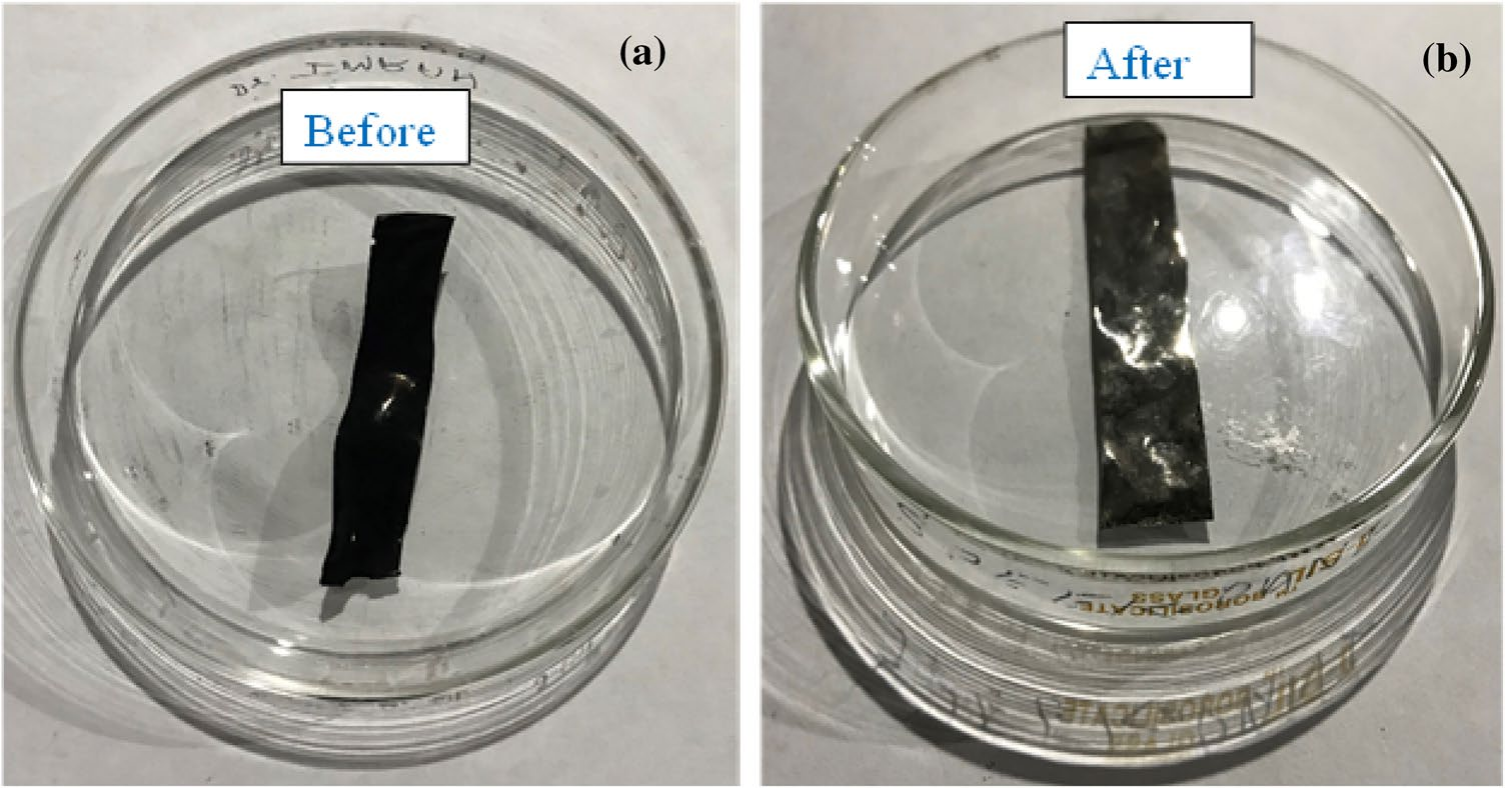
The fabricated membrane before and after coating with PANI and Pt.

The ionomeric material as prepared above was cast into the petri dish covered with Whatman filter paper No. 1 for slow solvent evaporation at 60 ºC in a thermostatic oven. After complete drying, the polymer material was removed from the plates with the help of a spatula. The prepared polymer material was crosslinked by heating at 100 ºC for 1 h in a thermostatic oven. The black-in-color ionomeric membrane was found mechanically stable and suitable to carry out the actuation studies. After that, the material was transferred on the Petri dish for the slow drying of the membrane at 45 ºC overnight. By carefully using forceps, the membrane was shifted to the thermostat oven at 150 ºC for 1 h. Next, the dry membrane had undergone into in-situ polymerization by dipping the membrane into aniline monomer, slowly adding 60 mL K_2_S_2_O_4_ solution, and was covered with aluminum foil. To ensure complete polymerization, the reaction temperature was maintained between 0 and 10 ºC for 24 h. The fabricated membrane before and after coating with PANI and Pt are shown in Fig. 1a, b, and a few properties are mentioned in Table 1.

**Table 1:** Condition of preparation and ion exchange capacity of SA/SPVA composite cation exchanger material.

S. no	Polyvinyl alcohol (g)	Silicotungstic acid (g)	4-Sulphophthalic acid (mL)	Appearance after drying	IEC (meq g^−1^ dry film)
1	4	1	1	Black sheet	1.6

### Determination of ion exchange capacity (IEC) of ionomeric material

IEC of the SA/SPVA ionomeric material was evaluated via classical titration method^9,10^. The pre-weighted ionomeric material was immersed in 1 M HNO_3_ for 24 h to convert it into H^+^ form, then neutralized with distilled water and dried at 45 ºC. After that, ionomeric material was cut into small pieces and packed into a glass column. The ionomeric material was converted into Na^+^ form by passing 1 M NaNO_3_ through the column with a very slow flow rate (0.5 ml min^−1^). The dissociated H^+^ ions were then titrated with 0.1 M NaOH solution using phenolphthalein as indicator^29^. The IEC value of the ionomeric material in meq g^−1^ dry material was calculated using the formula given below, and mentioned in Table 1;

1 $${\text{Ion}}\;{\text{exchange}}\;{\text{capacity}} = \frac{{{\text{Volume}}\;{\text{of }}\;{\text{NaOH}}\;{\text{consumed }} \times {\text{Molarity }}\;{\text{of}}\;{\text{ NaOH}}}}{{{\text{Weight}}\;{\text{ of}}\;{\text{ the}}\;{\text{ dry}}\;{\text{ membrane}}}}$$

### Electroless plating of the membrane

The ionomeric membrane of SA/SPVA was plated using the previous reported method. In short, the membrane of size (1 × 3 cm^2^) was treated with an aqueous hydrochlroic acid solution followed by the addition of tetraamineplatinum(II) chloride monohydrate [Pt(NH_3_)_4_Cl_2_·H_2_O] and ammonium hydroxide for 5 h at room temperature. The excess unreacted platinum was removed by washing the membrane using distilled water. The platinum ion was converted into platinum metal by treating membrane using an aqueous solution of sodium borohydride. Finally, the membrane was washed using distilled water and converted into H^+^ ion form using an aqueous solution of hydrochloric acid. The platinated membrane was stored in a desiccator and used for further studies as and when required.

### Water absorption capacity

The water absorption capacity of SA/SPVA/Pt ionomeric membrane was determined at room temperature for 1.5, 3.0, 4.5, 6.0, 7.5, 9.0, 15.0, and 20.0 h as discussed in literature^30^. In short, the pre-weighed dried membrane was immersed in the distilled water for the time period mentioned above, and the membrane was removed from water blotted quickly with Whatman filter paper and weighed again. The water absorption capacity was calculated using the formula given below:

2 $$W = \frac{{\left( {W_{wet} - W_{dry} } \right)}}{{W_{dry} }} \times 100$$

where *W*_*dry*_ is the weight of dry membrane and *W*_*wet*_ is the weight of water absorbed membrane.

### Loss of water with voltage

The pre-weighed platinated ionomeric membrane, i.e., SA/SPVA-PANI-Pt having the highest water absorption**,** was subjected to electric potential of 3–6 V for a different time interval from 4, 8, 12, and 16 min. The weight after applying the potential concerning time was quickly recorded. The loss of water for the membrane subjected to electric potential was calculated using the formula given below:

3 $$Water\,loss \% = \frac{{W_{1} - W_{2} }}{{W_{1} }} \times 100$$

where W_1_ and W_2_ are the weight of wet membrane and weight of membrane after the loss of water after applying the voltage.

#### Effect of voltage on the current density of the membrane

The pre-weighed platinated ionomeric membrane with the highest water absorption was subjected to electric potential of 1–6 V using linear sweep voltammetry method of potentiostat/galvanostat. The voltage and current data generated was recorded.

#### Determination of proton-exchange capacity of the membrane

The proton-exchange capacity of the platinated ionomeric membrane (breadth 1 × length 3 cm^2^) having the highest water absorption was determined by an impedance analyzer connected to potentiostat/galvanostat as per the procedure described in detail by various research groups^23^. The proton conductivity (σ) was calculated as follows:

4 $$\sigma = \frac{L}{R \times A}$$

where σ is proton conductivity in (S cm^−1^), L is the thickness of membrane in (cm), A is the cross-sectional area of polymer membrane, and R is the resistance.

#### Bending Actuation performance of the ionomeric membrane

The ionomeric membrane having the highest water absorption was subjected to the voltage in the range of 0–6 V, and the bending performance of the membrane was observed.

## Characterization of the film

### FTIR analysis

**Figure 2:**
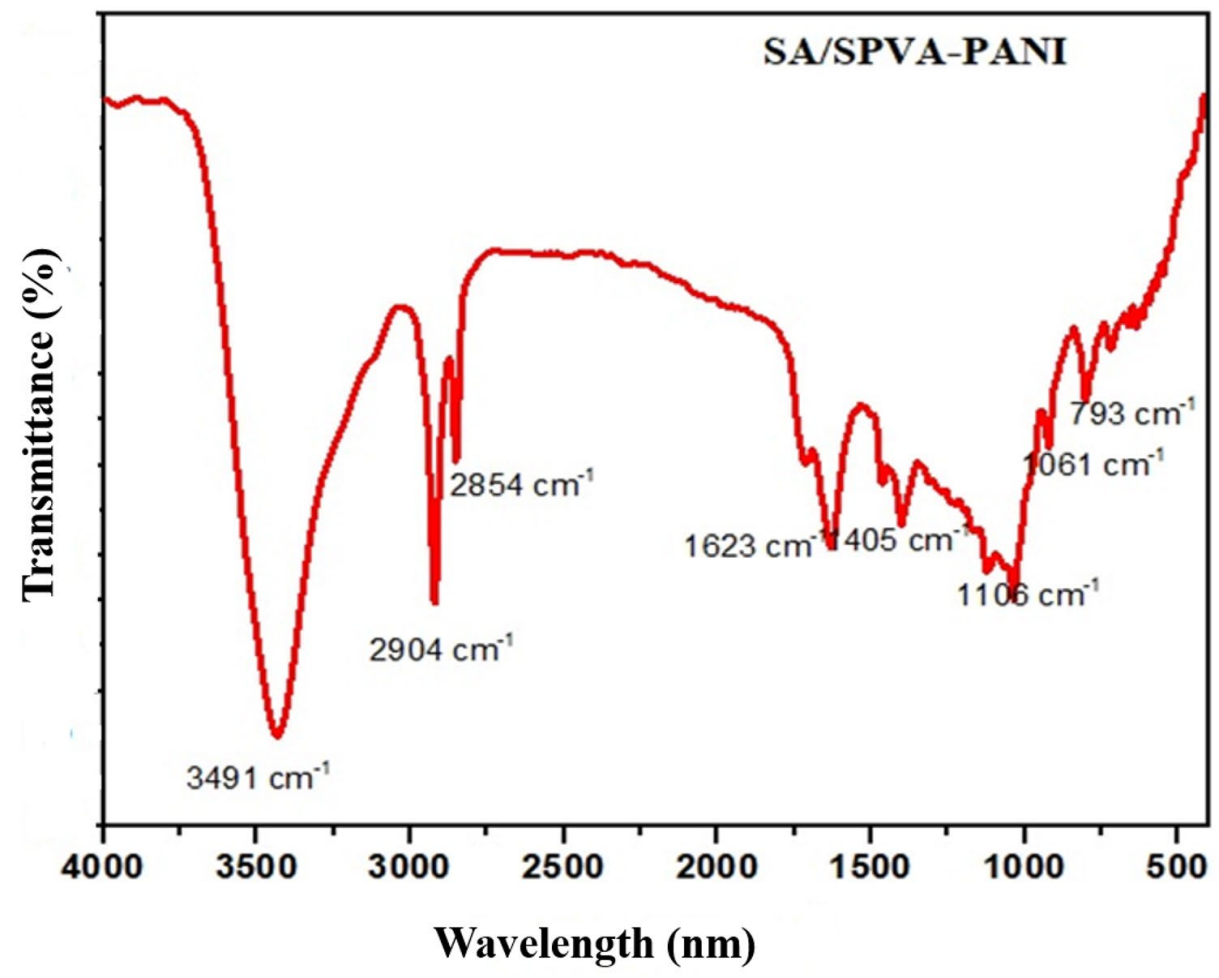
FTIR spectrum of the synthesized membrane SA/SPVA-PANI.

The FTIR spectrum of the synthesized membrane (SA/SPVA/PANI) was taken in the range of 4000–400 cm^−1^ to investigate the characteristic vibrations of various functional groups present in the ionomeric material. The spectrum shown in Fig. 2 revealed the vibration peak at 3491 cm^−1^ which corresponds to –OH stretching vibrations attributed by the alcoholic group of polyvinyl alcohol (PVA). Moreover, the vibration of the carbonyl group (CO) is also reflected in the spectrum at 1623 cm^−1^^31^. Furthermore, the vibrations around 2904 cm^−1^ and 2854 cm^−1^ reflect the -CH stretching due to the CH_2_ and CH_3_ groups of the PVA. Additionally, the vibrations correspond to 1405 cm^−1^, 1106 cm^−1^, and 1061 cm^−1^ are displayed in the spectrum attributed to quinoid and benzenoid rings of the benzene ring of PANI^32^. Also, the vibration at 793 cm^−1^ can be ascribed to the Si–O stretching vibration of silicotunsgtic acid^33^.

### XRD analysis

**Figure 3:**
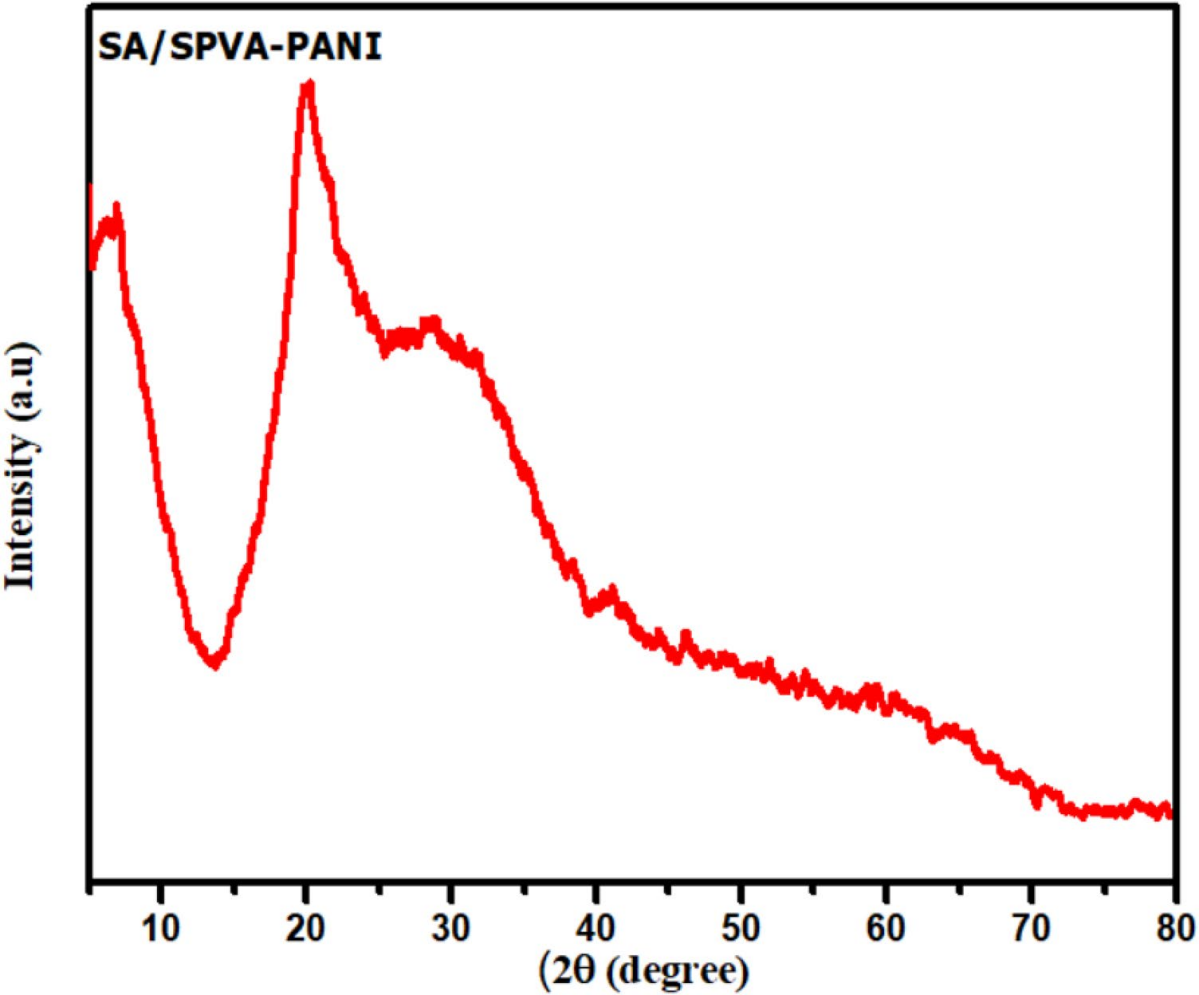
Shows the XRD pattern of the synthesized membrane SA/SPVA-PANI.

The XRD patterns obtained from the prepared membrane are shown in Fig. 3. The XRD patterns represented diffraction peak at 2θ values in which one at ca. 20.8° is the characteristic peak of poly(vinyl alcohol)^34^. Moreover, the broadening of a peak in the region 20° to 25° indicates the presence of polyaniline in the synthesized composite. In comparison, no peaks of sulphonic acid (SA) were found in the given spectrum. This may be a consequence of the amorphous nature of PANI that may have suppressed the peak of SA. This XRD investigation shows the semi-crystalline nature of the SA/SPVA-PANI membrane due to the polymeric nature of PANI^35^.

### SEM analysis

**Figure 4:**
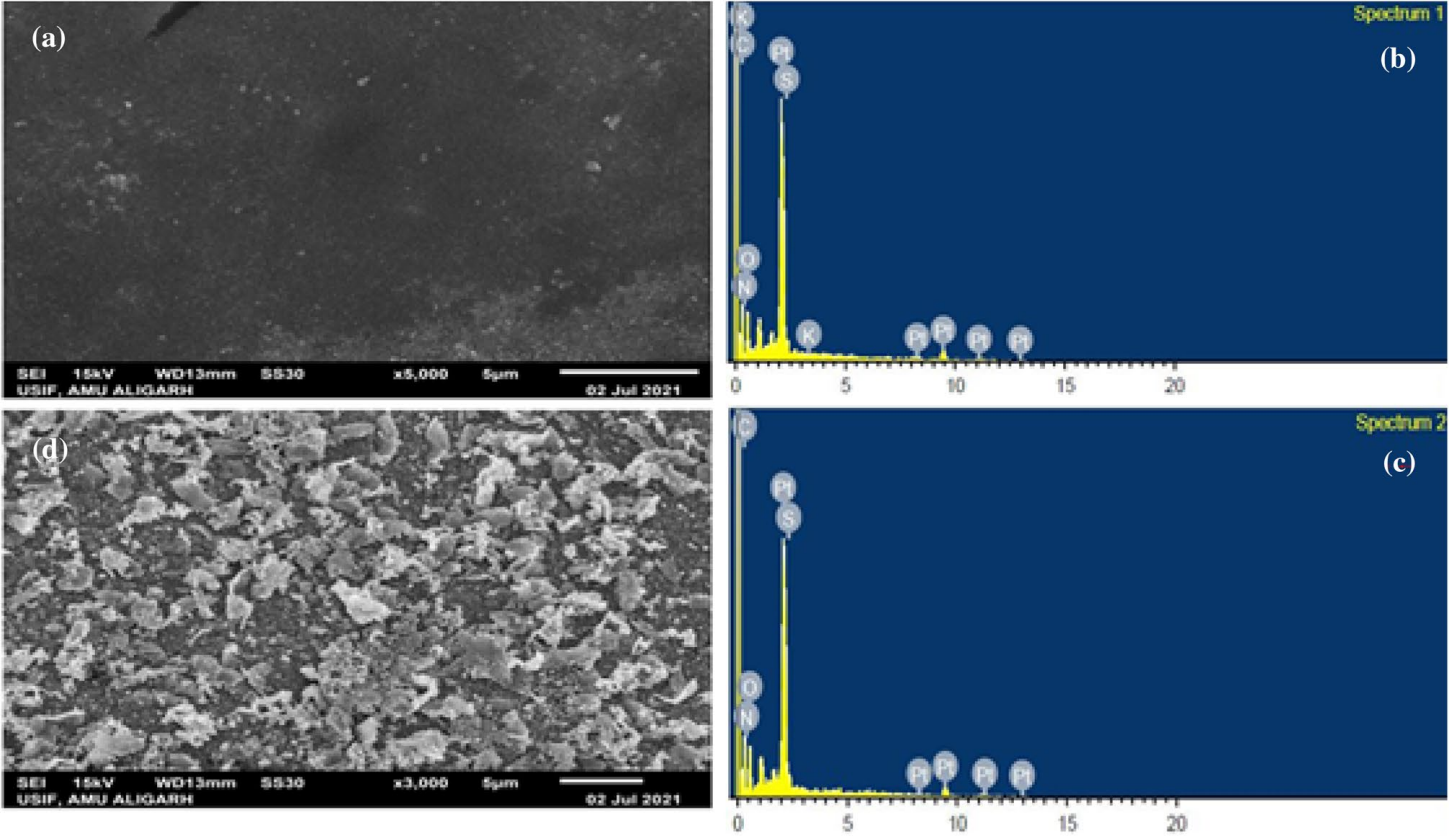
Represents the SEM and EDX images of the fabricated membrane (**a**) and (**c**) (SA/SPVA-PANI-Pt). (**b**) and (**d**) EDX images (SA/SPVA-PANI-Pt).

The surface morphology of the synthesized membrane SA/SPVA-PANI was carried out using scanning electron microscopy (SEM). The SEM micrograph, as shown in Fig. 4a has a porous and rough texture which is due to the combination of silicotungstic acid with PVA and PANI.

As can be seen in the image, the porosity of the ionomeric material is due to the presence of inorganic moiety (silicotungstic acid), which improves the membrane's ion exchange capacity. Furthermore, the open network of the polymer backbone (PVA) and the PANI served as conducting channels that improved the access of the ions through these pores, consequently, expected to enhance the performance of the synthesized membrane. In addition to this, the SEM micrograph was taken after the platinum coating is shown in Fig. 4b which reflects smooth texture along with the homogeneity on the surface that may enhance the conductivity of the synthesized membrane. Moreover, the SEM image was coupled with the elemental dispersive x-ray spectroscopy (EDS) before and after coating to figure out the elements present in the synthesized membrane. As can be seen, Fig. 4c, d are displaying almost all the elements present in the membrane such as carbon (C), oxygen (O), Sulphur (S) and tungsten (w) and Pt after coating. Notably, Potassium is also seen after coating, which may be due to the K_2_S_2_O_8_ solution used during the synthesis of the membrane.

From this analysis, it can be concluded that membrane synthesis has been done successfully.

## Results and discussion

**Figure 5:**
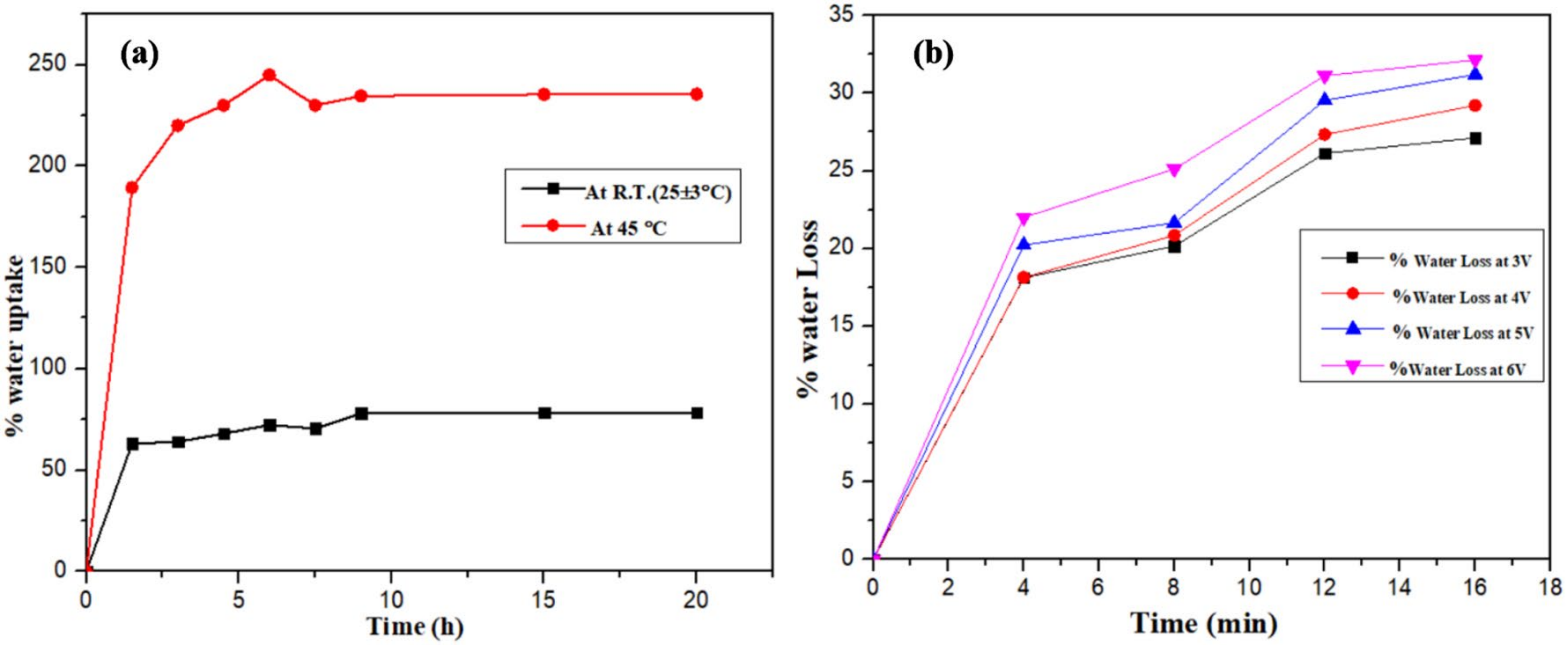
(**a**) Percentage water uptake of the SA/SPVA-PANI membrane and (**b**) percentage water loss of the SA/SPVA-PANI membrane at room temperature.

The ionomeric material SA/SPVA was prepared by the sol–gel precipitation method. The sulphonation of PVA was carried out by using sulfophthalic acid. The ion exchange capacity of the ionomeric material was found to be 1.6 (meq g^−1^ dry membrane).. The prepared ionomeric material synthesis is relatively easy, and the properties of this ionomeric material are comparable with that of the commercially available fluorinated ionomeric material, especially Nafion, and thus, it is expected to be a good substitute for the Nafion for bending actuator applications. Therefore, the as prepared ionomeric material is suitable for the fabrication of bending actuator as a replacement to the quite expensive fluorinated commercially available ionomeric materials. The water absorption capacity of the ionomeric material is potentially important for the better actuation performance of the bending actuator. Various studies showed that the movement of ions responsible for the bending of the ionomeric membrane is achieved in the hydrated form of the ionomeric membrane. It is well understood that the higher is the water absorption capacity of the membrane the better is the movement of the hydrated cations, and hence, the better bending actuation. The ionomeric membrane was subjected to water absorption at different time intervals. It was observed that the membrane absorbed about 63% water within 1.5 h immersion of membrane into the water, which was gradually increased to 70% after 7 h (Fig. 5a). Further, a slow absorption was noticed of about 8% and increased to 78% after 9 h with a constant trend till 20 h, showing that membrane got fully hydrated just after 9 h. The membrane having the highest absorption capacity, i.e., membrane socked in water for 9 h, was selected for further studies.

As discussed above, it is noticed that the membrane hydration is one of the important factors responsible for the better actuation performance of the ionomeric membrane. The absorbed water from the membrane is generally lost naturally, through rupturing of the membrane and electrolysis. When membranes are subjected to voltage to achieve the actuation, the electrolysis on the membrane surface occasionally occurs, leading to the evaporation of the absorbed water which may be one of the reasons for the short life of the ionomeric membrane. The water loss for the ionomeric membrane SA/SPVA-PANI-Pt having the highest absorbed water subjected to the different voltages (3 to 6 V) for 4–16 min (Fig. 5b). It was observed that the water loss from the membrane increases with the increase of the applied potential. The minimum water loss was observed at 3 V as the external stimulus. Therefore, 3 V of applied potential was considered appropriate for actuation performance of the ionomeric membrane.

**Figure 6:**
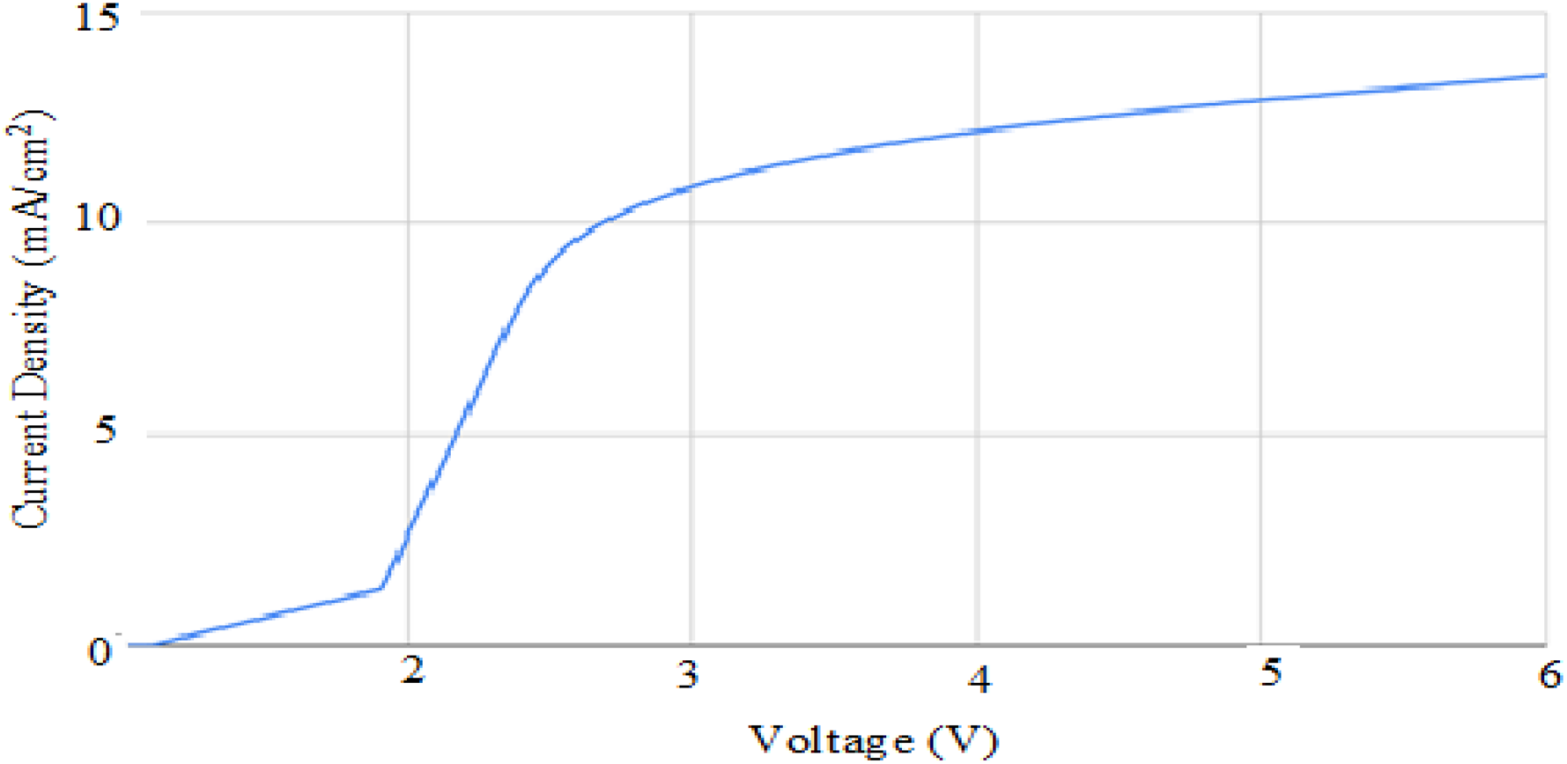
Current density vs voltage for the selected membrane.

The ionomeric membrane was also subjected to the linear potential from 0 to 6 V, and the current generated was observed, and represented in Fig. 6. The results showed that the observed current density of the membrane increases significantly with an increase in the potential up to ca. 3 V; ca. 10.5 mA cm^−2^ of the current was achieved. However, after that, it increased slowly and gets almost saturated until 6 V, proving a maximum current density of ca. 14 mA cm^−2^. The bending actuation was proposed to achieve a high value at an applied potential of 3 V. However, the higher current density at elevated potential showed that current flow is sufficiently high for bending. Still, the loss of water beyond 3 V may somehow decrease the bending performance of the ionomeric membrane.

**Figure 7:**
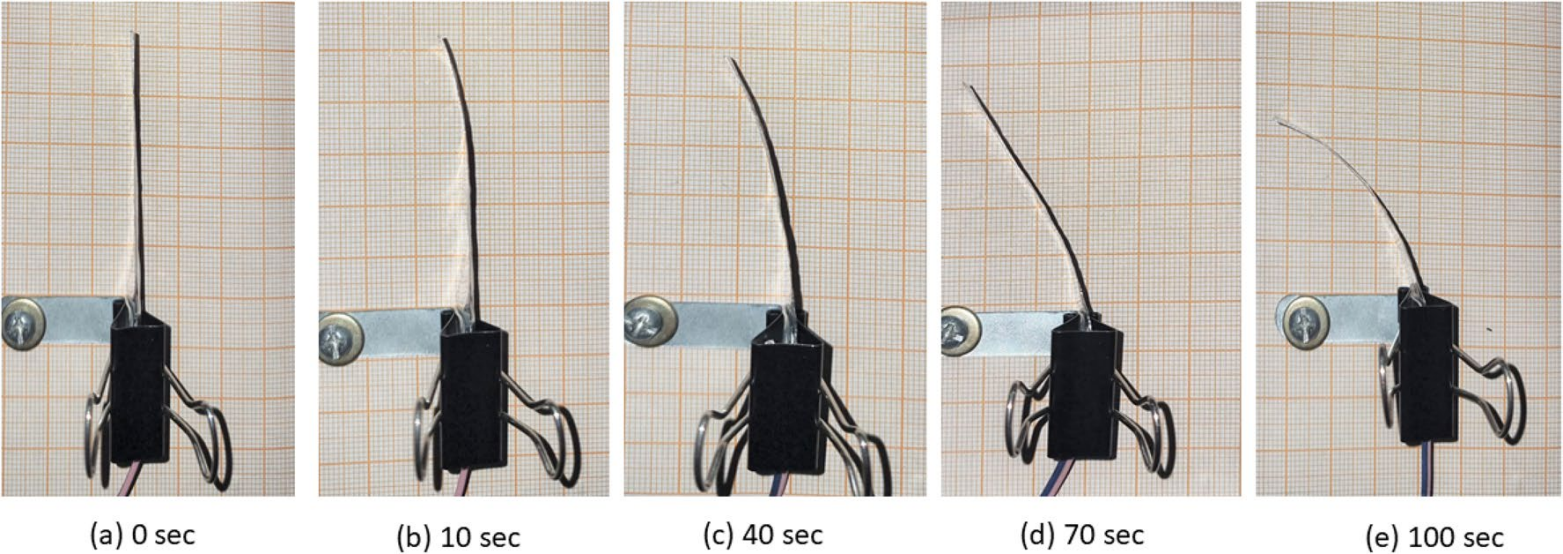
Experimental tip displacement response of SA/SPVA-PANI-Pt membrane with applied voltage of 3 V at 0, 10, 40, 70 and 100 s.

In contrast to ion exchange capacity, the proton exchange capacity is also one parameter for the better performance of the ionomeric membrane. This ionomeric membrane showed an appreciable proton exchange capacity of 1.9 × 10^−3^ S cm^−1^. The high proton exchange capacity of the ionomeric membrane is responsible for a good performance of actuation. The bending actuation of the ionomeric membrane was achieved after applying 3 V for different time intervals (10–120 s). The tip displacement data is tabulated as given in Table 2 and presented in Fig. 7. The results showed that the ionomeric membrane showed a maximum tip displacement of 32 mm within 100 s. The results of bending actuation are comparable with the similar ionomeric members already explored in the literature^8,36^.

**Table 2:** Deflection response of the SA/SPVA-PANI-Pt membrane with applied voltage of 3 V.

S. no	Time (s)	Deflection reading (mm)
1	10	2
2	20	6
3	30	11
4	40	13
5	50	18
6	60	20
7	70	23
8	80	28
9	90	30
10	100	32

## Conclusion

In this study, SA/SPVA-PANI-Pt composite cation exchange membrane was fabricated by solution casting method. The excellent ion exchange capacity 1.6 meq g^−1^ of, water holding capacity of 425%, proton conductivity 1.9 × 10^−3^ S cm^−1^, and a fast actuation capability were obtained by the synthesized membrane. This membrane has an excellent water retention limit along with minimum water loss when voltage was applied. In addition, quick actuation is achieved by tip relocation parameters. Consequently, the synthesized membrane can be effectively used for micro/soft-robotic actuation purposes. This will pave the way for new opportunities in the highly dynamic and quickly evolving field of micro/soft robots.

## Data Availability

All data generated or analyzed during this study are included in this published article.
